# Systematic Observation of an Expert Driver's Gaze Strategy—An On-Road Case Study

**DOI:** 10.3389/fpsyg.2017.00620

**Published:** 2017-04-27

**Authors:** Otto Lappi, Paavo Rinkkala, Jami Pekkanen

**Affiliations:** ^1^Cognitive Science, University of HelsinkiHelsinki, Finland; ^2^Traffic Research Unit, University of HelsinkiHelsinki, Finland

**Keywords:** naturalistic tasks, observational instruments, eye tracking, fixation classification, gaze coding, eye movements, driving, expertise

## Abstract

In this paper we present and qualitatively analyze an expert driver's gaze behavior in natural driving on a real road, with no specific experimental task or instruction. Previous eye tracking research on naturalistic tasks has revealed recurring patterns of gaze behavior that are surprisingly regular and repeatable. Lappi (2016) identified in the literature seven “qualitative laws of gaze behavior in the wild”: recurring patterns that tend to go together, the more so the more naturalistic the setting, all of them expected in extended sequences of fully naturalistic behavior. However, no study to date has observed all in a single experiment. Here, we wanted to do just that: present observations supporting all the “laws” in a single behavioral sequence by a single subject. We discuss the laws in terms of unresolved issues in driver modeling and open challenges for experimental and theoretical development.

## Introduction

This paper takes a slightly unusual, even unorthodox, approach to studying car drivers' visual behavior. Observations of an extended behavioral sequence of just one highly experienced driver are used as a case study of the rich and varied strategies experienced subjects employ in natural tasks.

Even the apparently simple task of driving down a winding country road involves sophisticated gaze information processing and behavior (at this point wish to take a peek at the **Supplementary_Movie_1_full_video.mp4** which we will be analyzing in this observational study). It is only the ease with which humans are able to routinely perform such tasks belies their underlying complexity. This complexity is starkly revealed in artificial intelligence and robotics (e.g., the design of autonomous cars), where real-time interaction with complex 3D environments has turned out to be one of the most daunting tasks for a machine to perform. Much of the complexity is not well revealed, however, by highly simplified laboratory tasks, where a subject is asked to maintain fixation on a “fixation target” or pursue a “pursuit target” (typically small dots or geometrical shapes on a blank background) or search for a “feature conjunction target” among distractors. None of these tasks reveals the dynamic interplay of gaze, action and the spatial world. Yet the active strategies of natural behavior is something we need to understand if we are to understand vision—for if we do not understand how humans actively sample and organize the information available in rich naturalistic stimuli, we do not understand the visual input to the brain (Ahissar and Assa, 2016; Lappi, 2016).

Based on the data available in the literature—collected in different experiments with different tasks and presented from the point of view of different theoretical interests—it can be difficult to get a “feel” for the behavioral patterns. Especially so for researchers who are not themselves involved in eye tracking, and have not accumulated the type of tacit knowledge that comes from working with raw data. There are two main reasons why this is particularly true in eye tracking. One is that the *quantitative* patterns are still most often given using very coarse *aggregate measures* such as the horizontal dispersion in “visual search” (e.g., Underwood et al., 2002) or area of interest dwell times aggregated over a “trial” (e.g., Foulsham et al., 2011). This type of aggregate representation tremendously simplifies statistical analyses, but does not reveal the role individual fixations play in the underlying strategies—what information might be gleaned from the fixations, or how they support the on-going behavior. Yet work on natural task gaze strategies has consistently shown that individual fixations focus with high specificity on targets most relevant to the task (Land, 2006; Tatler et al., 2011). This adaptive character of individual fixations is rendered unobservable when the eye movements are not looked at the level of fixations, but instead aggregated into scalar variables such as horizontal gaze direction variability, AOI gaze catch %, fixation counts or saccade counts. The other reason is that *qualitative* descriptions of “what the participants were looking at” are usually given only *verbally*, or with the help of a single still image from a gaze video (e.g., Figure 13 in Land, 2006; Figures 4, 5 in Wann and Wilkie, 2004). Neither way of representing the observations is conducive to giving the reader an overall understanding or the dynamical aspects of the phenomenon: how gaze target selection, and more generally gaze-interaction with the complex natural settings, evolve over time.

One of the goals of this study is, therefore, to show the richness of natural gaze behavior in an active real-world task—driving. Driving is in many ways an ideal domain to study real-world locomotion. For one thing, it is easier to instrument a car than a pedestrian for reliable measurement. Also constraining limb actions to steering wheel and pedal movements in itself brings about a useful reduction in degrees of freedom, the road environment is typically more stereotypical and simple in layout than most of our locomotor surroundings. Finally, the driving task can be studied at all levels of expertise from driving school students to professional drivers, with most of the adult population in modern countries potential test subjects somewhere in between.

Driving, and the way we use eye movements to sample the spatial world from behind the wheel, are also often invoked as an important real-world application of models of attention, perception, or memory—at least in introductory vignettes (e.g., Regan and Gray, 2000; Wolfe and Horowitz, 2004). Kowler (2011) in reviewing 25 years of research into “how eye movements cope with real world visual and cognitive demands” identified driving (along with tasks such as reading, and sports) as a core task the understanding of which would reveal much of interest about how the visual system works.

Driver eye movements have been investigated for decades, in both real world driving (e.g., Shinar et al., 1977; Land, 1992; Land and Lee, 1994; Green, 2002; Lappi et al., 2013b; Lehtonen et al., 2014) and driving simulator experiments (e.g., Wilson et al., 2007; Mars, 2008; Mars and Navarro, 2012; Lemonnier et al., 2015). Yet, if we want to understand driver gaze behavior in terms of where people look (the identity of each fixation target) and when (how they sequentially allocate gaze time to multiple parallel targets), then the extensive literature actually presents a somewhat fragmented picture of specific tasks and experimental settings, but no “overall story.” The main goal of this paper is to describe and portray in their natural ecological context strategies that have been revealed in quantitative experimental work. We use prior experimental work to systematize our qualitative analysis: eye movements are fleeting and difficult to pick up and codify through observation with the naked eye. We therefore organize our observations of the gaze behavior of our expert subject by organizing them in terms of seven typically recurring aspects of gaze control in naturalistic tasks—the “seven qualitative laws of gaze behavior in the wild” (Lappi, 2016)—which have been observed severally in a wide variety of tasks, but to date never yet all together.

### “Seven qualitative laws” of natural gaze behavior in the wild (and their application to driving)

Eye tracking in naturalistic tasks has begun to reveal recurring patterns of gaze behavior that turn out to be surprisingly regular and repeatable. Based on, and extending, previous reviews (Regan and Gray, 2000; Hayhoe and Ballard, 2005; Land, 2006; Kowler, 2011; Tatler and Land, 2011; Tatler et al., 2011) seven “qualitative laws” of gaze behavior in the wild were identified by Lappi (2016). (L1–L7, Table 1) These “laws” were defined as recurring patterns that tend to go together, the more so the more naturalistic the setting, all of them expected in most extended sequences of fully naturalistic behavior.

**Table 1:** **General gaze strategies in naturalistic tasks, and how they are manifested in driving**.

**Seven qualitative laws**	**Examples in driving**	**Notes**
L1. Gaze patterns are highly repeatable and stereotypical	The way the driver scans the road surface (i) visual orientation to the curve apex during curve approach and turn-in (sometimes called “tangent point orientation,” Land and Lee, 1994) (ii) optokinetic nystagmus (OKN) elicited during cornering (Authié and Mestre, 2011; Lappi and Lehtonen, 2013; Lappi et al., 2013b; Itkonen et al., 2015).	Stereotypy is found within and between individuals, given task constraints and physical context.
L2. Gaze is focused on task-relevant objects and locations	Scanning intersections, traffic, potential hazards etc. (e.g., Lemonnier et al., 2015). In curve driving, gaze concentration in a fairly small visual region in the view of the road ahead (some 10° horizontally, 5° vertically) (e.g., Land and Lee, 1994; Lappi et al., 2013b; Lehtonen et al., 2014).	Top-down control, rather than the visually most salient ones repeatedly “capturing” gaze.
L3. Individual fixations have interpretable functional roles	In the curve driving literature steering models attempt to account for how *Guiding Fixations* to various “steering points” may produce visual input for different control mechanisms (Land, 1998; Salvucci and Gray, 2004; Boer, 2016; for reviews see Wann and Land, 2000; Lappi, 2014). See also *look-ahead fixations* (L5) and Results and discussion for their possible role(s).	The roles are not always intuitive. The pattern of gaze can often be surprising to the subject as we are usually relatively unconscious of our eye movements, and cannot report them verbally.
L4. If possible, targets are fixated “just in time” (Ballard et al., 1995) 4.1. In complex tasks gaze is tightly coupled to the information requirement of the imminent subtask 4.2. Typically gaze leads action by about 1 s	In driving, the gaze-to-steering time delay is typically about 1–2 s (Land, 1992; Chattington et al., 2007; Land and Tatler, 2009), and the time headway typically about 2 s (Lappi and Lehtonen, 2013; Lehtonen et al., 2014). Lead time is the delay from gaze shift toward the bend to steering input. Time headway is the path distance from current location to the point of fixation divided by speed. These properties have been used to define operationally *guiding fixations* in driving (Lappi and Lehtonen, 2013), cf.L3 above.	“Just in time” means the moment they become relevant for guiding the next action, in contrast to than scanning the targets well ahead of time (requires cognitive resources for maintaining information in short-term memory, and carries risk the obsolescence of that memory). Unless a(sub)task requires continuous monitoring/tracking, gaze disengages—i.e., switches to a new target—before (sub)task completion.
L5. Visual sampling is intermittent^i^ 5.1. In sequential tasks “just–in–time fixation” (or guiding fixations) are interleaved with look-ahead fixations 5.2. In dual-task performance guiding fixations (and look-ahead fixations) are interleaved with fixations to targets relevant to a parallel task	Look-ahead fixations have been identified in approach to a bend (Lappi and Lehtonen, 2013; Lehtonen et al., 2013, 2014, cf. gaze polling in Wilkie et al., 2008). Glancing at the instruments can be interpreted as another form of intermittency, i.e., gaze time shared between tasks (Johnson et al., 2014).	In most tasks, we do not “stare” at a single target for a prolonged period of time, but we instead sample the visual world with fixations lasting typically 100–500 ms interspersed by rapid, intermittent, saccades when visual input is degraded and actively suppressed by the visual system (see e.g., Land and Tatler, 2009). Visual information is not available to the brain continuously but as fairly discrete samples.
L6. Memory is used to (re)orient in 3D space	Long-term memory contribution to driving has been little studied, but Shinoda et al. (2001) show that traffic signs are more reliably noticed when they occur in locations that would be expected by prior knowledge of the traffic system than unexpected locations. Land and Tatler (2001) discuss the visual strategy of an expert racing driver in terms of a rich memory representation of the lap, and the possibility of orienting gaze, head and the vehicle in a way that takes into account road geometry beyond the range currently in view.	This can be done even to targets currently outside the field of view, implying trans-saccadic spatial memory (Tatler and Land, 2011). Also the fact that there are few fixations to irrelevant objects (i.e., visual search) implies stable contextual representation of where the task–relevant objects and locations are in 3D space (cf. e.g., Land et al., 1999).
L7. Gaze control is always part of “embodied” eye/head/body/locomotor control	On the one hand, gaze shifts are achieved by rotating not only the eyes but also rotating and translating the head and the body. On the other hand, head and body movements in space are compensated for by gaze–stabilizing eye and head rotations. For the brain, scanning and fixating targets and changing one's point of vantage are not necessarily separate “modular” tasks where the total output (gaze) would be a linear sum of separate locomotor, head and oculomotor systems (see discussion in Steinman, 1986; Collewijn et al., 1992; Lappi, 2016).	One shortcoming of visual steering models as interpretations of fixation behavior (L3) is that they only formalize the steering response as a function of visual information that is assumed to be available through appropriately coordinated gaze behavior (Donges, 1978; Salvucci and Gray, 2004). I.e., they do not discuss how fixation behavior itself is organized as part of a general gaze-steering strategy (but see Land, 1992; Land and Furneaux, 1997).

i *This intermittency formulation is more general than the guiding fixations/look-ahead fixations formulation in Lappi (2014) and Lappi (2016). Guiding fixations and look-ahead fixations are defined in terms of visual requirements of different phases of a single ”task”. Sharing gaze time on the other hand happens ”between tasks”. But that definition presupposes an a priori delination of task strucutre, which in naturalistic tasks is not trivial. In driving, say, is operating the vehicle to be understood as one task, and monitoring traffic (via mirrors) another task (gaze time sharing between tasks)? Or should mirror-checking be interpreted as preparation for subtasks, such as a lane change (GF/LAF within a task)? Or is even interleaving guiding and look-ahead fixations in curve negotiation to be understood as sharing gaze time between control and anticipation tasks? The distinction seems more semantic than substantial, at least unless a highly rigorously specified task model is available*.

However, to date they have been observed singly or a few at a time in tasks as various as making tea (Land et al., 1999), making a sandwich (Hayhoe et al., 2003), drawing from a model (Land, 2006), steering a car (Land, 1992; Land and Lee, 1994; Lappi et al., 2013b) and a number of sports such as tennis (Ripoll and Fleurance, 1988), cricket (Land and McLeod, 2000; Mann et al., 2013), and squash (Hayhoe et al., 2012). That is, no empirical study (or review) to date has exhibited these common recurring patterns in a single task. Thus, to bolster the claim (Lappi, ibid.) that *all* these patterns would be expected to be present in extended sequences of fully naturalistic behavior we wanted in the present study to present observations of all the “laws” in a single behavioral sequence of a single subject. We use driving as our behavior of choice, following and building on the analysis in Lappi (2014).

We next proceed to describe the observational methodology used in this study. Then, in the Results and Discussion section we analyze the gaze patterns qualitatively, i.e., in terms of how and what they reveal about the seven qualitative laws of gaze behavior in the wild. We close with a discussion of open issues and challenges to existing perceptual-cognitive and control theoretical driver models arising from observing real-world behavior in context.

## Methods

### Participant

The subject was a 43 year old male licensed driving school instructor with 25 years of driving experience and 18 years of experience in professional driver education. He was recruited by personal contact. He had normal uncorrected vision and a valid driver's license. He reported no medical conditions that might affect eye movements.

### Ethics statement

This study was carried out in accordance with the recommendations of Finnish Advisory Board on Research Integrity. The protocol was approved by the ethics committee of the Faculty of Behavioral Sciences, University of Helsinki. Written informed consent in accordance with the Declaration of Helsinki was obtained from the participant. This was done in the form of a fixed-format consent form explaining the purpose of the study, the procedure, and intended use of the data (publication of anonymous data for scientific purposes). A paper copy of the consent form was archived.

### Test site, equipment and procedure

The test road (Velskolantie, Espoo: N 60.273951, E 24.654733) was a 5.13 km low-standard two-lane rural road (5.5 m pavement width, painted edge lines) with low traffic density. The test vehicle was a MY 2001 Porsche Typ986 3.2 (trade name “Boxster S”) with a manual transmission (Dr. Ing. h.c. Ferdinand Porsche AG, Stuttgart, Germany). The car was not familiar to the driver, but as he was an expert with a wide experience of operating different vehicles he displayed no apparent difficulty with the controls, adapting immediately. The eye tracker was a Pupil Labs Binocular 120 (Pupil Labs UG haftungsbeschränkt, Berlin, Germany). The headset has a forward-looking world camera with an approximately 100° (horizontal) by 56 degrees (vertical) field of view, and two eye cameras. The sampling rate for the eye cameras was set to 30 Hz. The Pupil software with in-house custom code ran on an ASUS Zenbook UX303LB 2.4 GHz, with Linux Debian 4.2.6. and kernel 4.2.0. A custom built headband was used to secure the headset more firmly.

Upon arriving at the test site, the participant was briefed on the procedure, after which he filled the informed consent form. The driver was shown the test route on a map, and explained that the instruction was simply to drive the route “as they normally would.” After adjusting the driving position, the eye-tracker was calibrated, and the calibration accuracy was immediately checked by the same 15-point procedure (see below). The researcher operating the eye tracker (PR) in the passenger seat gave instructions at crossroads leading to and from the test route proper. There were no intersections or crossroads on the test route. The road was run in both south-north and north-south directions. A post-calibration was then performed allowing us to determine calibration accuracy and also to improve it offline in post-processing.

### Eye tracker calibration

**Figure 1:**
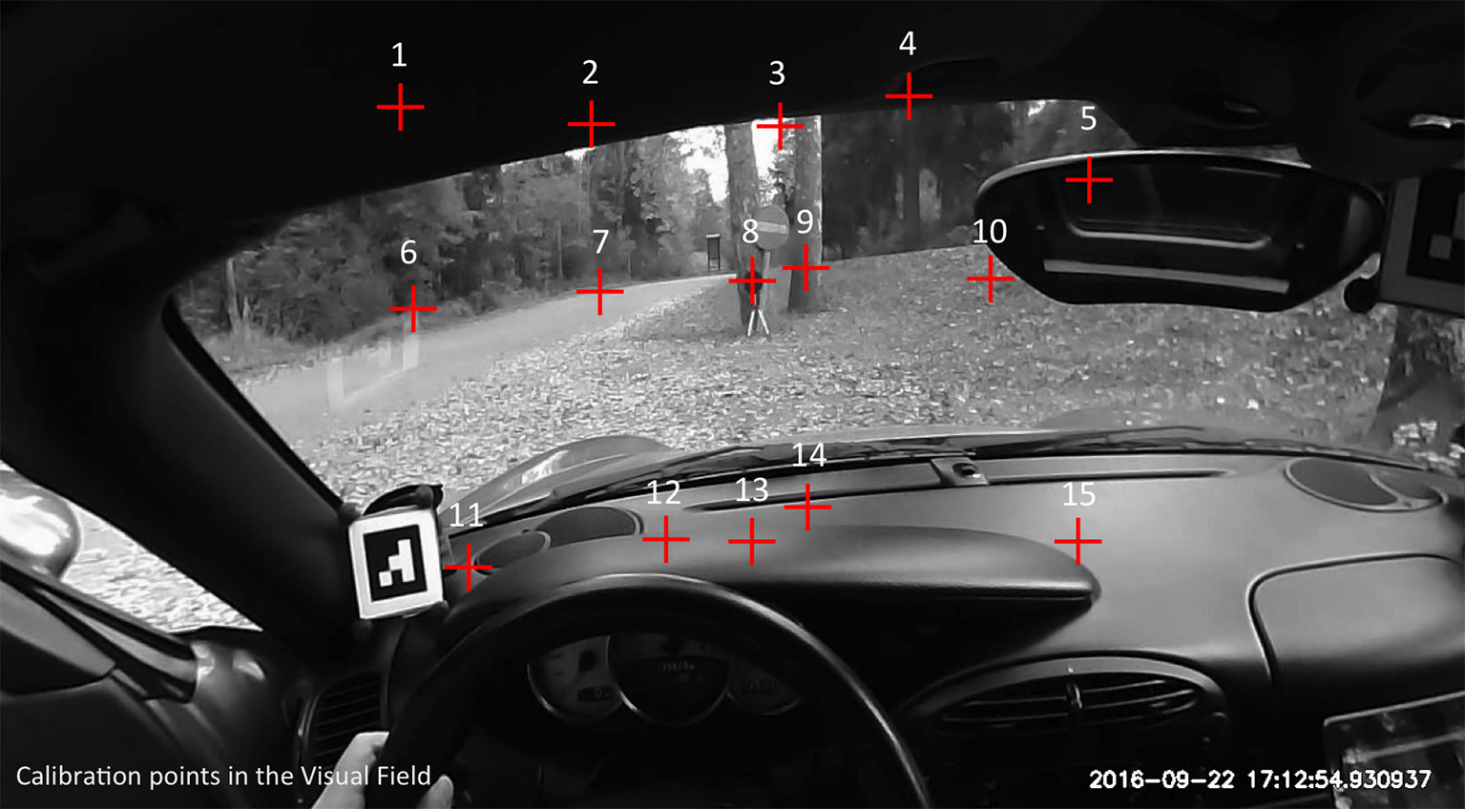
**Locations of the calibration points in the (head centered) visual field**. Note that instead of multiple targets, we used a single target (on the tripod, at fixation point 8). The participant was instructed to adopt different head poses that moved the target to different locations in the visual field.

The eye tracker was calibrated using 15 points in the visual field (Figure 1). Note that rather than presenting targets at 15 physical locations, a single target (about 5 m in front of the vehicle) was used, and the participant was asked to adopt different head poses, moving the target to different parts of the (head-referenced) visual field. Extensive pilot testing was done to arrive at a protocol whereby the instructions are clear and natural to the participants and they can follow them in an efficient and repeatable way. While this method does not give us *complete* control of the positioning of the target locations in the field of view, it nevertheless has a number of advantages. From a practical point of view, it does not require a large and cumbersome 15-point calibration frame to be transported—a single target on a tripod suffices. Second, the target can be placed at a large distance (rather than, say, on the vehicle bonnet or even at an arm's length inside the cockpit), thereby reducing parallax error.

### Post-processing

In post-processing camera lens distortion was corrected using OpenCV v.2.4.9.1. CV2 undistort tool. Calibration stability was checked manually, and where it was deemed bumps or headset movement had shifted the calibration, it was adjusted manually. These adjustments were small, and mainly to the vertical coordinate. Time stamp (ts) of each frame based on Unix time stamp was burned into the video. This produced the final gaze-overlay video for analysis.

The video was inspected visually in slow motion for recurring typical fixation patterns, and an iterative method was used to arrive at a codification of gaze targets with the following desiderata: (i) It should be as free as possible from any specific theoretical or functional interpretation, that is, it should not be confined to any specific theoretical point of view in the literature on driver eye movements but accommodate all, (ii) the classification should allow for a reasonably unambiguous classification of all fixations in the video, (iii) the classification should give a good balance between categorizing *all* fixations in an *informative* way, but with as few categories as possible. (iv) the fixation classes should be mutually exclusive, that is, each fixation should be categorizable into one and only one class. This classification was the basis for our General Observations that are intended to characterize the overall pattern in the present gaze data (presented in Section General observations).

After this initial rough classification was in place, a more detailed analysis of episodes most relevant to the core task of steering the vehicle was done. Specific bends were selected on the basis that they should contain sufficient variability and detail to allow meaningful discussion of “the seven qualitative laws of gaze behavior in the wild” (see Section Introduction). The selected episodes were annotated, marking the beginning and end point and a putative classification of each fixation using a custom video annotation tool (https://github.com/jampekka/scvideonaxu). The rule used was that for a sequence of gaze positions to qualify as a fixation, gaze should remain stable at a fixed position or a fixed target object or location for a minimum of three frames (~90 ms). This putative classification was then discussed and refined in debriefing sessions within the research group to come up with a final classification. Episode videos were then prepared that display the annotations overlaid on the video (Supplementary Movies 2–5), serving as basis for more detailed illustrations of the more general “the seven qualitative laws” [presented in Section Detailed description of selected episodes (illustrations of the “qualitative laws”)].

## Results and discussion

We find it first useful to *show* the entire gaze video to give the reader a “feel” of the dynamical characteristics of gaze behavior on the road (rather than just individual frames and descriptions of the *spatial* fixations locations, or time *series* gaze data without the physical context). The full video is given as supplement (**Supplementary_Movie_1_full_video.mp4**). Observing this video will give the reader an idea of the richness and complexity of natural behavior even in a fairly controlled setting with little traffic, no intersections, no expansive vistas etc. The reader familiar with, say, the driver modeling literature and eye tracking experiments can from here get a feel for the gaps in present experimental work and models, which may hopefully inspire development of future experiments and models.

However, eye movements are fleeting and because we have poor conscious access to our own eye movement behavior it may be difficult to develop intuitions and identify the patterns through untutored observation with the naked eye. Here prior experimental work and models can, conversely, be useful in providing a framework for interpreting the observed behaviors, rendering “observable” behaviors that could otherwise be easily missed.

We will first present general observations about the overall pattern of how the driver scans the scene (in terms of a rough fixation classification), on the entire behavioral sequence. In the next section we will look in more detail at some “episodes,” behavioral subsequences, which we consider to reveal interesting phenomena when looked at in light of the seven “qualitative laws.” In both cases we present still images as figures, and verbal descriptions in the main text, to give the reader anchoring points and communicate our interpretations. But we urge the reader to consult the videos given as Supplementary Material.

### General observations

The first and perhaps most striking features are the high frequency of gaze shifts (gaze lability) and the amount of head movements. These are the features most people almost immediately and unprompted have remarked on when they have seen the video, as we are usually not *aware* of this lability of our eyes, and the frame in which they are supported (the head). This is because when the head and eye rotation are *actively controlled*, the perceptual system knows the motor command send to the eye/head system (efference copy), and can predict and thereby take into account sensor motion in creating a stable percept and maintaining orientation (for discussion see Angelaki and Hess, 2005; Burr and Morrone, 2012; Ahissar and Assa, 2016).

**Figure 2:**
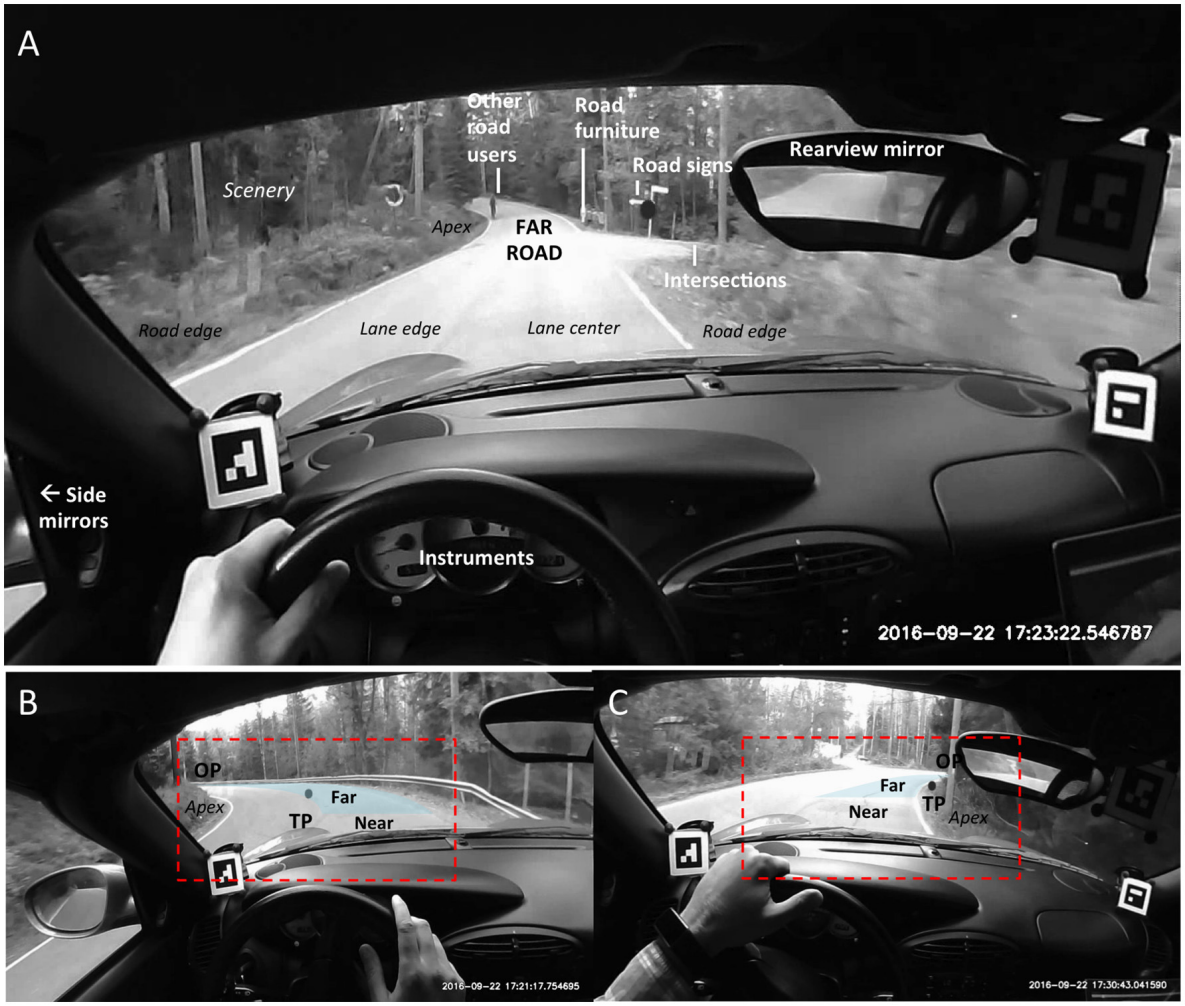
**(A)** Descriptive classification of fixations used in this study, shown here in representative video frames on a straight. **(B,C)** Illustrate The Far Road “triangle” in a left turn **(B)** and a right turn **(C)**. See also Figure 4. OP, Occlusion Point; TP, Tangent Point. See main text for explanation.

It is clear from the video that our driver never “stares” at any particular location or object for any extended period of time. Instead, the entire scene is scanned all the time with the rapid saccade–fixate–saccade pattern characteristic of visual (and) manual exploration. Remember that the participant is a driving school instructor: this behavior is consistent with the instruction given in Finnish driving schools to continually “rotate the gaze.” Figure 2 gives an overview of the “scanning pattern.”

The fixations could be partitioned into different categories in a number of ways. One rather natural classification scheme is given in Table 2 (cf. Figure 2). We use this classification to organize our general observations (G1–G7) of how each of the seven target classes figure in the overall scanning pattern.

**Table 2:** **Overall fixation target classification and general observations (G1–G7) about the video**.

**Fixation target class**	**General observation**
The (Far) Road	G1. The driver tends to keep his eyes on the (far) road, unless other relevant targets present themselves, and always quickly returns to it.
Instruments and Mirrors	G2. Instruments and mirrors are checked regularly.
Road users (traffic)	G3. Other road users in view are monitored, often with repeated fixations.
Intersections (side roads)	G4. Side road intersections are usually checked with a sideways glance.
Road Signs	G5. Most road signs are checked with a sideways glance.
(Other) Road Furniture	G6. Other road furniture is occasionally checked with a sideways glance.
Scenery	G7. “Scenery” not otherwise specified is rarely fixated.

#### G1 the driver tends to keep his eyes on the (far) road, unless other relevant targets present themselves, and always quickly returns to it

After scanning for other targets, gaze always returns to the road ahead. From inspecting the video it is clear that in terms of dwell time the (far) road would be the predominant gaze category. Note that the gaze almost exclusively seeks out the “far” road region, as opposed to the road immediately in front of the car. This is consistent with the two-level/two point control models (Donges, 1978; Land, 1998; Salvucci and Gray, 2004; Boer, 2016) that are based on the assumption that experienced drivers use gaze to obtain visual *preview* of road geometry used for *anticipatory* control (matching the *predictable* road curvature), as opposed to near road information for *compensatory* control (maintaining lane position against *unpredictable* perturbations). That there are very few fixations to the near road does not mean by any means that the driver would not be using visual information for stabilizing control, though. Indeed, it has been shown an experienced driver can monitor near road information peripherally (Summala et al., 1996), freeing the experienced driver to allocate more attention and overt gaze to the far road region than a novice (cf. Mourant and Rockwell, 1972; Lehtonen et al., 2014).

Of course, this behavior is also consistent with driving instruction frequently exhorting beginning drivers to try to look far enough ahead. But how far is far? There is no specific distance or time distance in the literature that would define “far” vs. “near” road. Typically, time headway to the “far” region in bends assumed to be about 1–2 s (cf. Lehtonen et al., 2014), which fits well with modeling literature as well (Boer, 2016).

In bends, it is conventional to use the *lane edge tangent point* (TP) as the point for segregating “near” and “far” road space (Land, 1998; Salvucci and Gray, 2004; Lappi, 2014). This is the point on the inside of the curve at which the visual orientation of the lane edge reverses its direction in the driver's visual field (Figures 2B,C). Note that the TP is a *travel point* (i.e., a point that moves with the observer in the 3D scene frame of reference, even though it may sometimes remain stationary in the observer's egocentric frame). Actual distance and time distance to the TP in the 3D world is therefore variable, and how far is “far” thus depends on curve geometry (time distance also depends on driving speed).

**Figure 3:**
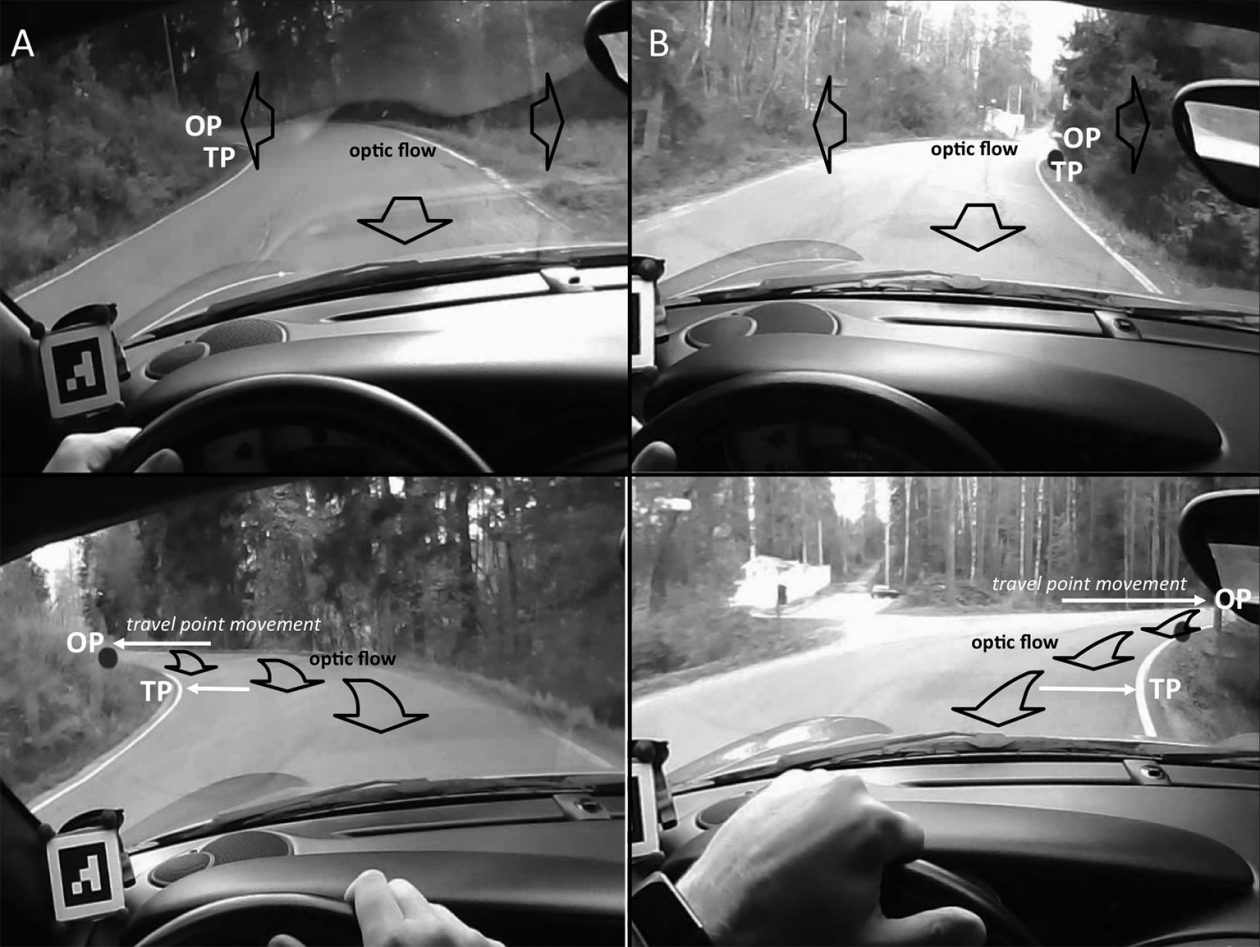
**Schematic illustration of how a bend “opens up” as the Occlusion Point travels up the road (and horizontally in the visual field), revealing more of the road. (A)** Left hand bend. Top panel: approaching. Bottom panel: turning in. **(B)** Right hand bend. Top panel: approaching. Bottom panel: turning in. The Occlusion Point (like the tangent point) is a *travel point*, not a fixed 3D location in the scene. Travel point motion in the visual field (indicated by the white arrows) does not match the optic flow (indicated by the black block arrows). Fixating a travel point may be a tracking fixation, achieved with a pursuit movement. The same is clearly true also for tracking stationary fixed 3D scene objects or locations, but here the tracking will match optic flow. OP, Occlusion Point; TP, Tangent Point.

Observing the video, we can see that in simple bends the TP and Occlusion Point (OP) together with the lane edge opposite to TP create a Far Road “triangle” (Figures 2B,C). For a good portion of the time, this gives as good qualitative characterization of where we look when “we look where we are going” on the road^1^. The *Occlusion Point* (OP) is the point furthermost part of the road to which a continuous, unobstructed preview of a possible trajectory (future path) is visible (Lappi et al., 2013a), i.e., the point where “the road disappears from view.” Like the TP, OP also is a *travel point*; it moves ahead as the observer travels along the road and does not follow the local optic flow (Figure 3; clear illustrations are e.g., 17:28:16.79–17:20:28.15 and 17:30:40.29–17:30:46.02).

#### G2 instruments and mirrors are checked regularly

The driver's visual field does not only cover the external 3D roadspace, of course, but also relevant targets in the vehicle frame. These include the instrument panel (speedometer, tachometer) and mirrors (rear view and side mirrors). These are scanned predominantly (but not exclusively) on straights (e.g., 17:30:46.02–17:31:06.26; 17:27:57.13–17:28:02.61), presumably because there is less task load than in curves (Tsimhoni and Green, 2001), and less need for visually monitoring the road ahead. Stabilizing steering control requires little overt gaze, and the much longer time headways to visual occlusion means that there is much less time pressure for spotting hazards.

#### G3 other road users in view are monitored, often with repeated fixations

Oncoming vehicles, or pedestrians/bicyclists coming the other way and being overtaken are monitored by fixations. (e.g., 17:22.09–17:22:16.53; 17:29:06.83–17:29:10.96 [cars emerging from blind bends]; 17:32:57.75–17:33:05.49 [coming up on two bicyclists simultaneously]).

#### G4 side road intersections are usually checked with a sideways glance

Whenever there is a side road or a road from a yard that intersects the road, the driver tends to scan it with a fixation or often multiple fixations (e.g., ts 17:20:34.56–17:20:40.79 [side roads on both sides of a straight]; ts 17:22:08.55–17:22:09.02 [sideways glance to a side road on the left side of a right hand bend]).

#### G5 most road signs are checked with a sideways glance

Road signs are fixated—even from quite impressive distances. These include road signs proper (*speed limit* signs *poor road surface* and *bends* caution signs, *stop* sign), as well as street name and navigational instruction signs (e.g., 17:20:17.47–17:20:21.52 [speed limits, bends]; 17:20:25-48–17:20:28.40 [bumpy road]; 17:34:08.52–17:34:25-72 [multiple]).

#### G6 other road furniture is occasionally checked with a sideways glance

Post boxes and other mid-sized objects near the road are occasionally “checked out” (e.g., ts 17:24:37.57–17:24:38.45). Here the driver is likely using high-spatial-resolution foveal vision for detailed analysis and object recognition of a target already *localized* and *individuated* as distinct from the ambient background using peripheral vision.

#### G7 “scenery” not otherwise specified is rarely fixated

There are actually very few fixations at “scenery” not covered in the above categories. This is in itself important. The *absence* of any significant number of fixations on the general scenery, that is, the concentration of gaze on specific target and *the apparent absence of any visual search* in itself indicates that *peripheral visual information is used in a very efficient way to guide the gaze at the relevant locations with high accuracy and reliability*. Note that we therefore prefer to use the terms visual *exploration* or *scanning* rather than visual *search*, see further discussion below.

In sum, the general pattern of gaze coordination is the following: First, the default gaze mode is “eyes on the road,” or “looking where you are going.” Second, glances elsewhere are performed when *a specific relevant target* to look at has been identified (also, there must be “spare capacity” to allocate gaze time to non-steering related targets).

### Detailed description of selected episodes (illustrations of the “qualitative laws”)

We next illustrate “the seven qualitative laws” by selecting specific episodes from the extended behavioral sequence for more detailed fixation-by-fixation observation. To recap, the “laws” are:

L1. Gaze patterns are highly repeatable and stereotypical.
L2. Gaze is focused on task-relevant object and locations.
L3. Individual fixations have interpretable functional roles.
L4. If possible, targets are fixated “just in time.”
L5. Visual sampling is intermittent.
L6. Memory is used to (re)orient in 3D.
L7. Gaze control is always part of “embodied” eye/head/body/locomotor control.

We will go through them in order, pointing out in each case relevant observations in the present data, open issues in the experimental modeling literature and deeper theoretical connections among the laws that may be non-obvious.

#### L1 repeatable and stereotypical gaze patterns

With a case study approach cannot tell from the data alone whether any stereotypy observed is idiosyncratic to the participant. But when we find repeatable patterns that are reported in the literature we can consider them general.

**Figure 4:**
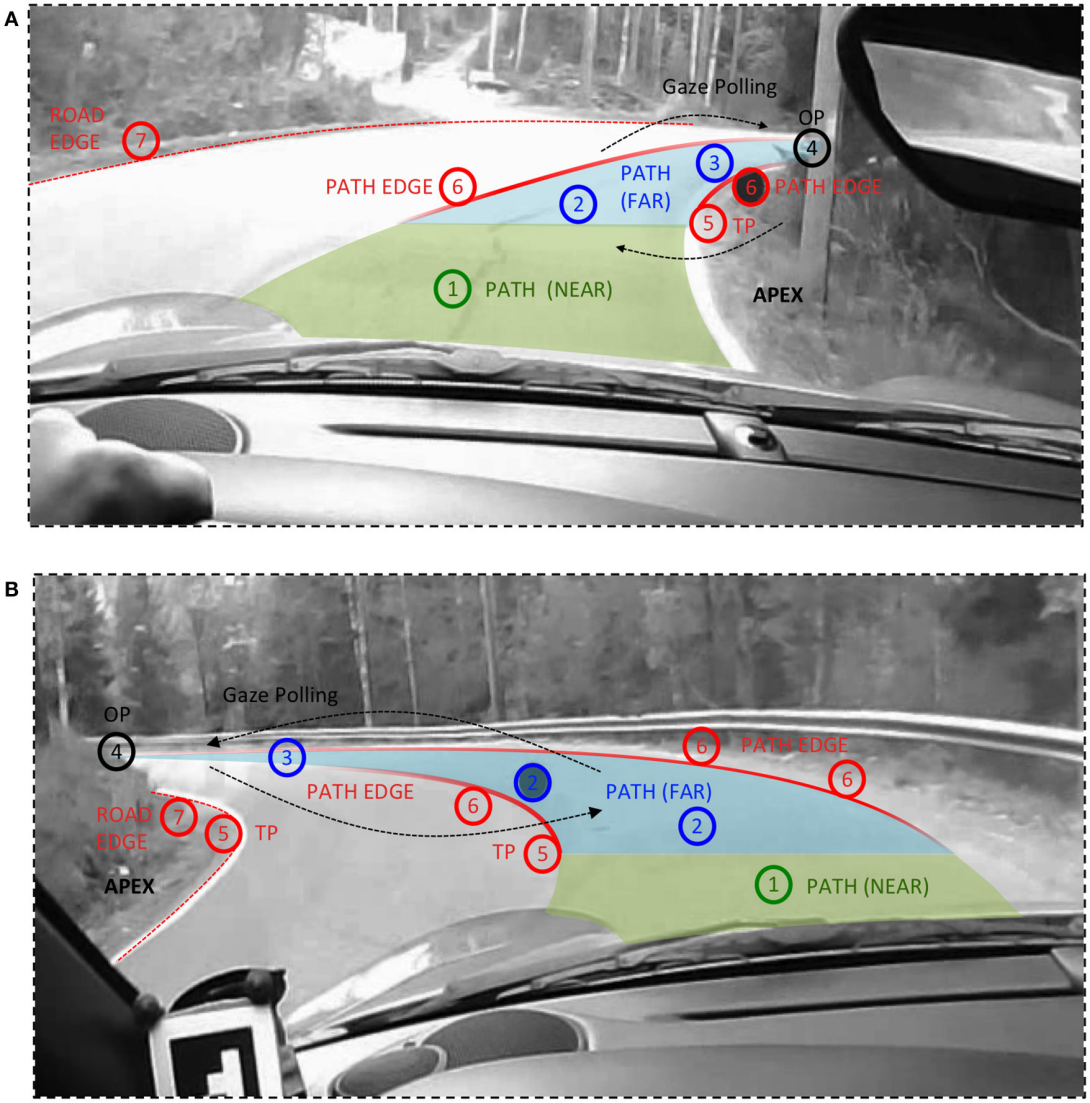
**Labeling of fixations at “the road ahead” in curve driving**. NB. These underlying road images are the insets from Figures 2B,C. Far Road (blue) is defined as driver's own lane beyond the tangent point distance. Near Road (green) extends from in front of the vehicle to the tangent point level. (1) Path (near), (2) Path (far), (3) Path (look-ahead), (4) Occlusion Point (look-ahead), (5) Path Edge (tangent point), (6) Path Edge, and (7) Road Edge (tangent point).

*Scanning the Far Road in bends*. Perhaps the most robust coordination pattern is the fairly systematic scanning of the Far road (Figures 2, 4) in bends. As the Occlusion Point travels up the road, revealing more of the scene behind (Figure 3), the gaze seeks out the road surface/inside road edge emerging into view.

This orientation toward the inside edge of an upcoming bend (the *apex* region) is sometimes called “tangent point orientation.” But when one observes the actual scan pattern, it should clear there is much more complexity involved than the driver aiming to stabilize gaze on a single point. What does the full picture of scanning the far road in bends look like, then? *TP* orientation occurs, especially in the *approach* and *entering* phases of the bend (Land and Lee, 1994), but there are also fixations to lane edges further ahead, beyond the tangent point, and to the road surface in the Far Zone region. These *guiding fixations* (GF) are interspersed by saccades to make *look-ahead fixations* (LAFs) further into the bend and beyond. When the view up the road is occluded, as is the case on this road which runs through woods, some of these LAFs reach the OP (see Figure 4), but not all. As the driver enters the bend, fixations of the TP and the inside lane edge beyond the tangent point, and fixations to the road surface in the Far Zone, mainly beyond the tangent point continue, interspersed by LAFs further up the road.

Because of this rich pattern and multiplicity of gaze targets, it is better to reserve the term *tangent point orientation* to fixations at or very near (within 3° of) the tangent point itself, performed at the very end of the approach and beginning of the entry (i.e., straddling the turn-in). This is according to the definition in the original Land and Lee (1994) study^2^. As a methodological side comment, note that because of the visual projection of road geometry into the forward-looking visual field can bring these points very near to one another, making the definite determination of the actual gaze target of many individual fixations difficult, and traditional AOI methods unreliable (Lappi et al., 2013a; Lappi, 2014). But while in many individual cases the classification of a given single fixation could be ambiguous bases on instantaneous gaze position alone, the overall pattern of multiple gaze targets in the Far Zone is clear.

In the general case, due to the highly dynamic character of the way the road surface presents itself in the visual field (Figure 4), Far road fixations cannot be separated from LAFs by any hard-set distance or *gaze angle* criterion^3^. Instead, we propose that LAFs should be defined by the following *return* saccade. That is, in a LAF a fixation on the TP/far road/road edge is followed by a saccade further up the road (but not necessarily all the way to the OP), and a *return saccade* back to the road surface/lane edge closer to the vehicle (i.e., *gaze polling*, Wilkie et al., 2008). This “zig-zagging” pattern is very evident in most bends—for example the right hand bend in the beginning of **Supplementary_Movie_2_17_21_31_rl.mp4** and the left hand bend in **Supplementary_Movie_3_17_23_48_l.mp4**—and we have seen it to occur more or less frequently in the raw data of every driver we have ever tested in our previous studies.

#### L2 gaze focused on task-relevant object and locations

Although, the scanning patterns in driving are sometimes called “search” (primarily when complex situations call for identification and interpretation of multiple potential *hazards* e.g., Underwood et al., 2003; Wolfe and Horowitz, 2004), for the analysis of the core cognitive requirements of driving (high-speed vehicle control) we prefer the term *scanning* or visual *exploration*, as there is hardly any evidence of visual search proper—at least not in the way the term is used in experimental psychology. In visual search paradigms eye movements are used to look for a target among distractors, *where the* target is *masked* by the clutter and there is hence substantial *uncertainty over target location* (as in a typical Feature Integration Theory paradigm search matrix, or a Where's Waldo? image). The way the fixations appear to *immediately* find specific targets even at quite impressive distances (the road, other road users, traffic signs, see e.g., fixations to the three traffic signs in **Supplementary_Movie_2_17_21_31_rl.mp4**) suggests this is not the case in driving, as in fact we see very few fixations to “scenery” (general observation G7).

Intermittency combined with a high concentration of gaze on *relevant* targets (lack of search) implies efficient peripheral vision processes for target identification and saccade planning. Here we should bear in mind, though, that the participant is an expert driving school instructor, for whom traffic signs, for example, are highly relevant in terms of carrying out in-car instruction. Thus, he might have superior parallel covert “search” strategies compared to more typical drivers. Whether traffic signs are as “salient” to everyday drivers in (terms of being able spot them from distance with peripheral vision and “attracting” gaze) is not clear.

#### L3 interpretable functional roles for individual fixations

Let us return to the far road fixation targets in curve driving. How can the individual fixations in the scanning pattern be interpreted? This is an important question, because the reason most eye movement research focuses on *fixation* behavior is that fixation is considered functionally as the “window” when new visual information is available to the brain, punctuated by saccades during which relatively little information is received, and analysing where and when fixations are made is taken as a road to inferring underlying cognitive processes. We would like to point out here that for a moving observer, a fixation tracking a fixed target in the scene—and most travel points as well—is, from an *oculomotor* point of view, a pursuit movement (see discussion in Lappi, 2016).

In very broad terms, far road fixations can be considered simply “looking where you are going.” But *interpreting* this strategy—*why* we should look where we are going in the first place—needs to take place in terms of *how* the brain processes the information gleaned from the fixation(s), and uses it real-time control of gaze and locomotion. Overt behavior (gaze position) does not uniquely specify the information that might be gleaned, and many interpretations for the (guiding) far road fixations have been put forward (for review see Wann and Land, 2000; Land and Tatler, 2009; Lappi, 2014). A detailed discussion of all the different interpretations, and their underlying theoretical motivations and commitments, are beyond scope of this paper. References to key papers are given in Table 3. The fixation classification scheme (Table 3, Figure 4) is intended to be compatible with any and all theoretical interpretations, i.e., not committed to any particular theoretical viewpoint or interpretation.

**Table 3:** **Labeling used, and interpretations found in the literature, for fixations in the Far road**.

	**Fixation class**	**Characterization**	**Interpretations available in the literature**
1	Near path	The road surface immediately in front of the vehicle that the vehicle will imminently travel over (path).	*Compensatory control* in a two-level framework (Donges, 1978); *Near Point* in a two-point model (Salvucci and Gray, 2004).
2	Far path	The road surface ahead, in the bends at the tangent point level.	*Anticipatory control* in a two-level framework (Donges, 1978); *Far Point* in a two-point model (Salvucci and Gray, 2004). *Guiding fixations* on the *Future Path* (Boer, 1996, 2016; Wann and Land, 2000; Wann and Swapp, 2000; Wann and Wilkie, 2004; Wilkie et al., 2008).
3	Far path (look-ahead fixation)	The road surface further ahead, in the bends beyond the tangent point level.	*Guiding fixations/Look-ahead fixations* on the *Future Path* (Lehtonen et al., 2013, 2014). Gaze polling (Wilkie et al., 2008).
4	Occlusion point	The furthest point the road surface is continuously visible to.	*Look-ahead fixations*; trajectory planning and/or monitoring oncoming traffic (Lehtonen et al., 2013, 2014).
5	Tangent point (road/path edge)	Where the visual orientation of the lane edge reverses its direction.	Steering by the Tangent Point (Raviv and Herman, 1991; Land and Lee, 1994); *Far point* in a two-point model (Land, 1998).
6	Path edges	The edges of the driver's own lane in the far region, where they constrain the path the driver can choose.	Road geometry constraints on the *Field of Safe Travel* (Gibson, 1938); potential *Line Crossing* locations (Godthelp, 1986); *Safety Line* (Mars and Navarro, 2012).
7	Road edge	The edge of the opposing lane.	Potential *Line Crossing* locations?

*i.e., class G1 in Table 2. The numbers refer to Figure 4*.

Far Path targets (Table 3, class 2) are postulated in several steering models to be steering points, as is the tangent point (class 5). (While the travel points and waypoints on the path would instantaneously occupy the same location, they will generally move in different directions in the visual field, cf. Figure 3, they will be useful for quite different steering strategies, see (Lappi, 2014) for detailed discussion). Look-ahead fixations (classes 3,4), LAFs on the other hand are considered to be different from such guiding fixations, because the tight gaze-steering coordination needs to be uncoupled during a LAF. They may nevertheless support higher-level trajectory planning (see L6, below). Note that we have used the term Path Edge (classes 5 and 6), which we define as those parts of the lane edges, in the Far region, which at any particular moment in time constrain available paths. (We reserve the term *lane edge* for the *entire* edges of the driver's own lane, extending beyond these “path edge” regions—even beyond the current field of view ahead and behind the vehicle). Road edge (classes 5 & 7) refer to the edge of the opposing lane.

#### L4 targets fixated “just in time”

Driving is a self-paced task in that the driver has a choice in the speed s/he wishes to travel at. However, once a speed is chosen the targets emerge at a given pace and obsolescence rate (cf. Senders et al., 1967; Kujala et al., 2015). This places a high importance on the accurate *timing* of fixations and saccades.

At an aggregate level this temporal coordination is reflected in a robust ca. 1 s gaze-action delay. Chattington et al. (2007) report gaze lead time (peak of gaze-steering cross correlation) of 0.98 s for 60 s epochs. We have observed similar cross correlations on the same road as used in this study for seven participants (*unpublished data* from Lappi et al., 2013a).

At an individual fixation level, judgments of gaze-action delay depend on an interpretation of which action(s) each individual fixation actually supports. For steering related guiding and look-ahead fixations this question is still unresolved (cf. previous point). The most frequently referred to phenomenon remains the final fixation at or near the tangent point before turning into a bend (Land and Lee, 1994). This is clear for example in the blind right hand bend in **Supplementary_Movie_4_17_30_39_r.mp4**. However, given that the TP region is frequently fixated several times in anticipation (not just “just in time”), and that other locations in the Far Road Triangle are fixated in curve negotiation, the full picture of how *fixation timing* and *locomotor action timing* are related remains unresolved (cf. the next point).

The just-in-time strategy also implies the visual system needs to be able to recover either from peripheral visual information or from memory *where* the need-to-know information is at any moment in time. Cf. discussion of lack of search above (L2) and the role of memory below (L6).

#### L5 intermittent sampling

We remind the reader of the general observation that our driver never “stares” at any particular location or object for any extended period of time. Scanning the scene with the rapid saccade–fixate–saccade pattern happens all the time. That is, the overall pattern gives the intermittent “feel” that is characteristic of visual *exploration* (and exploratory behavior in other modalities, e.g., manual exploration).

Intermittency is clear for example in **Supplementary_Movie_5_17_30_55_lr.mp4** in the way looking “where you are going” is interspersed with fixations to a traffic sign and other road furniture, an intersection on the right, and the side mirror. Also in the right hand bend in the beginning of **Supplementary_Movie_2_17_21_31_r.mp4** where the fixations in the Far Road region are interspersed by look-ahead fixations and sideways glances at a traffic sign. What are the implications of such a sampling pattern for control and cognitive processing?

Visual steering control models in psychology (for a review see Lappi, 2014) and driver models in vehicle dynamics engineering (for a review see Macadam, 2003) generally do *not* address this intermittency in visual input (but see Johns and Cole, 2015 for discussion and one of few experimental studies investigating the effects of intermittency of visual input to steering control; cf. “active gaze” approach in artificial intelligence and mobile robotics, Ballard, 1991; Fermüller and Aloimonos, 1995). Rather, the current state-of-art approaches in sensing and control are typically “reactive” systems, i.e., the system passively receives continuous input and produces output control signals in response. In contrast, in psychology and cognitive science it's well established—and apparent also in the present data - that in active dynamic tasks humans “proactively” sample visual information as needed, leading to input that is *intermittent*, and determined by the active observer (e.g., via eye movements) rather than imposed by the environment as a “forcing function.” This allows humans to transcend their relatively slow information processing and limited sensory resolution to achieve impressively high aptitude in high-speed steering control (in driving and other domains).

On the other hand given that some relevant visual information may be available not *continuously* but as *discrete fixations*, critical action decisions may only be doable at certain points in time, or at least there are likely to be limited optimal “windows” for timing locomotor action initiation relative to oculomotor actions. This issue has been studied in e.g., sports psychology in the literature on the quiet eye phenomenon (Vickers, 2016); but so far it has not been studied in the driving domain, nor modeled in driving models, beyond the above mentioned general 1 s lead time (cross correlation) between “apex orientation” and steering.

#### L6 memory used to (re)orient in 3D

Memory processes cannot be readily “read off” from gaze behavior in our locomotor task—especially given that the route was only driven once (observing change in gaze behavior over multiple runs would be more informative of memory processes, as would analysis of landmark use in familiar surroundings, cf. Spiers and Maguire, 2007, 2008). Also note that the fact that in the present data even first fixations on a target are achieved without search, based on peripheral information (cf. discussion on L2, e.g., traffic signs) means that fixation-without-search in highly familiar surroundings (such as one's kitchen; Tatler and Land, 2011) cann*ot* be interpreted as proof of memory use. Also the just-in-time fixation strategy (L4) emphasizes the online nature of eye-hand-body coordination and “letting the world be its own model” (cf. Brooks, 1991)—as opposed to maintaining information in memory (which requires cognitive resources and faces the problem of that information becoming obsolete).

On the other hand memory and *intermittency* (L5) are deeply connected at a theoretical level, because it is to a large extent intermittency that makes memory (as opposed to pure online control) powerful. Anticipation allows humans to transcend their relatively slow information processing and limited sensory resolution. Modern cognitive theories of skilled action are predicated on the hypothesis that humans make predictions of the immediate future, choose actions on the basis of these predictions (for reviews of this *predictive* approach to anticipation and control, see e.g., Bubic et al., 2010; Henderson, 2017; for a critique of predictive control and defense of anticipation from merely *prospective* control see Zhao and Warren, 2015).

The relevant memory processes would be navigational long-term memory—an area of intense active research in the cognitive and computational neurosciences (Spiers and Barry, 2015). Integrating this literature to the theory of skilled driving would significantly advance out understanding of the driving task, and the role of these representations in real-world tasks generally.

Here we suggest one of the roles of look-ahead fixations—justifying the strategy of taking gaze time away from imminent needs of the primary control task—is to maintain and update this trans-saccadic memory of scene layout. That is, LAFS are relevant for steering (with a substantially higher delay than the 1 s for guiding fixations)—*both* for selection of, or parameter setting for, motor plans (updating “*inverse* models” in control theoretical terms), *and* creating a richer internal (*forward*) model of the state of the environment and the prediction of likely effects of action.

Precisely what *kind* of trans-saccadic memory underlies spatial orientation, and maintains our coherent experience of space (cf. Land and Furneaux, 1997; Tatler and Land, 2011; Burr and Morrone, 2012; Spiers and Barry, 2015) is an important but underappreciated issue in understanding (expert) driver behavior

#### L7 gaze control part of “embodied” eye/head/body/locomotor control

One of the first remarks a number of people have made upon viewing the video (**Supplementary_Movie_1_full_video.mp4**) is expressing surprise at how much the participant's head moves. We experience the world as stable, even when the platform from which we observe it is not static. To achieve this *visual stability* in mobile contexts the brain must be able to take into account, in very sophisticated ways, both controlled (active, predictable) head movement and (passive, unpredictable) perturbations (Angelaki and Hess, 2005; Tatler and Land, 2011; Lappi, 2016).

Compensatory eye movements (vestibulo-ocular and optokinetic responses) and compensatory head movement stabilize gaze stable against unpredictable perturbances. Eye- head coordination is, on the other hand, *guided* by top-down attentional processes when target motion is *predictable*. A target moving in the visual field is tracked (pursuit) and large gaze shifts (saccade) achieved in part by synergistically turning the head, not just rotating the eyes in their sockets.

Synergistic eye/head gaze shifts are most apparent in fixations to the side mirrors. Pursuit of roadside objects, on the other hand, is usually done with eyes only, even for high eccentricities. In contrast, pursuit of OP is accompanied by head rotation anticipating vehicle rotation—even though the eccentricity is small, the gaze rotation anticipating or guiding locomotor rotation seems to recruit also head rotation, suggesting it is not only stimulus eccentricity but also stimulus relevance to ongoing motor action that is important in eye–head coordination. Even saccades and eccentric pursuit of *non-locomotor* targets can be done without a substantial head component (i.e., the head is kept aligned with locomotor path, side roads are checked with a sideways glance, for example), whereas orienting to the locomotor path (presumably for preview guidance information) involves a substantial head component—even when the eccentricity of the locomotor target is small. That is to say: the head is tightly coupled to steering-related “guidance” but less tightly coupled from non-steering related “scanning”—the head/gaze coupling is also *intermittent*.

## General discussion

In this paper we present and analyse qualitatively an extended observational sequence where we have measured an expert driver's gaze behavior while driving on a real road with no experimental instruction. With this naturalistic task setting we hope to elicit typical behaviors this expert would use to cope with the complexity and ambiguity inherent in the real-world task of driving—within the limitations to the “naturalness” inherent in using an instrumented vehicle approach. This goal is heuristic^4^ : to identify patterns one can “see” in naturalistic settings, but have not yet been codified in experimental procedures and quantitative contexts.

Naturalistic (observational) and controlled (experimental) work should complement one another. On the one hand, controlled experiments run the risk of becoming too far abstracted away from the task constraints and behavioral strategies and patterns that actually make up the behavior of interest in the real world. Without real world observations complementing laboratory results, this may go unnoticed! Observing visual strategies in naturalistic real-world tasks can therefore provide important ecological validation to the design and results of lab experiments, or suggest ways to make laboratory designs more representative of real-world task settings. On the other hand, simply making notes of “where people look” does not produce good science. Using quantitative techniques to extend observational capabilities beyond those of the naked eye can allow one to record novel aspects of natural behavior. That is, quantitative measurement procedures that have been developed in experimental work can produce observational data for more qualitative analysis as well. At a more conceptual level, especially with eye movements which are so rapid and to which we have so little introspective access, prior laboratory/experimental work can be highly valuable, even essential, in coming up with the descriptive framework of concepts and procedures (the non-experimental observational *paradigm*, if you will).

We hope further systematic observational studies may be inspired by, and extend the results of, this one. This type of research is missing in the literature on naturalistic task gaze strategies but should be useful in moving between fully naturalistic settings and experimentally controlled tasks; in both directions and for mutual benefit. Of course, only controlled experiments will be able to reveal the internal workings of brain mechanisms—but at the cost of restricting the behavioral context to very restricted and often simplified tasks, and typically imposing artificial constraints whose effects on strategies may be unknown, yet substantial. We feel it is important to keep a balance between the goals of experimental rigor and faithfulness to the phenomena. Observational studies such as this one can also identify gaps in existing knowledge, e.g., by showing behavioral patterns in more detail, suggesting new data analysis procedures, or even completely new experiments.

## Author contributions

OL: conceived the study, wrote the first draft and revised the manuscript; OL and PR: designed and piloted the data collection procedure; PR: collected the data; OL, PR, and JP: analyzed the results, prepared the videos/figures and wrote the final draft; JP: contributed the analysis/data annotation tools.

## Funding

PR was supported by a personal study grant from the Henry Ford Foundation. JP was supported by, and Open Access publication costs covered by, the Finnish Academy of Sciences project MulSimCo (279905). The funders had no role in concept, preparation or decision to publish.

### Conflict of interest statement

The authors declare that the research was conducted in the absence of any commercial or financial relationships that could be construed as a potential conflict of interest.

## Footnotes

^1^Methodological note to researchers used to making Area of Interest (AOI) based analyses. As an AOI the Far Road Triangle would make more sense in characterizing the *general pattern* than for example the TP cantered circular AOI used to investigate tangent point orientation (Land and Lee, 1994; Lappi et al., 2013b). Note, however, that as with the distance and time distance of TP the *size* and the *shape* of this AOI will vary dramatically, making any statistical analysis of gaze catch percentages or dewll times problematic. (For the related AOI overlap problem of fixed-size AOI gaze catch percentage/dwell time analysis see Lappi et al., 2013a; Lappi, 2014). There appears to be no *straightforward* and *general* way to bring the traditional AOI method from controlled lab studies to dynamic tasks. Methodological innovation is called for—because of differences in the “design of the stimulus,” not just noise and sampling rate issues (for discussion see Lappi, 2015).

^2^Generalizing the term “tangent point orientation” to the entire scan pattern would be perhaps warranted if “steering by the tangent point” could be considered a *general* account of “where we look when we steer.” But when there are other theories, and the empirical picture is clearly more complex, this sort of terminological conflation is to be avoided.

^3^When the curve geometry and speed are fixed, such criteria can be used to operationally define LAFs in *that particular physical context* (e.g., Lappi and Lehtonen, 2013; Lehtonen et al., 2014).

^4^In the traditional philosophical rather than the computer science sense of the word: organized, systematic activity aiming at empirical *discovery* rather than empirical *justification* (e.g., Lakatos, 1980).

## Supplementary material

The supplementary movies are available via Figshare under a CC-BY license:

Supplementary Movie 1 Full Movie (1.37 GB) **doi: 10.6084/m9.figshare.4498466**.

Supplementary Movie 2 (22.56 MB) **doi: 10.6084/m9.figshare.4498613**.

Supplementary Movie 3 (12.62 MB) **doi: 10.6084/m9.figshare.4498619**.

Supplementary Movie 4 (11.51 MB) **doi: 10.6084/m9.figshare.4498622**.

Supplementary Movie 5 (23.52 MB) **doi: 10.6084/m9.figshare.4498625**.
